# Diet Quality and Micronutrient Intake among Long-Term Weight Loss Maintainers

**DOI:** 10.3390/nu11123046

**Published:** 2019-12-13

**Authors:** Rebecca W. Pascual, Suzanne Phelan, Michael R. La Frano, Kari D. Pilolla, Zoe Griffiths, Gary D. Foster

**Affiliations:** 1 Department of Food Science and Nutrition, California Polytechnic State University, 1 Grand Avenue, San Luis Obispo, CA 93407-0386, USA; rpascual@calpoly.edu (R.W.P.); mlafrano@calpoly.edu (M.R.L.F.); kpilolla@calpoly.edu (K.D.P.); 2 Department of Kinesiology & Public Health, California Polytechnic State University, 1 Grand Avenue, San Luis Obispo, CA 93407-0386, USA; 3 WW International, Inc., New York, NY 10010, USA; zgriffiths@ww.com (Z.G.); gary.foster@ww.com (G.D.F.)

**Keywords:** weight loss, weight loss maintenance, micronutrient intake, micronutrient deficiency, Healthy Eating Index, estimated average requirement, obesity

## Abstract

Inadequate vitamin and mineral intake is documented among individuals with obesity, but is unknown among long-term weight loss maintainers (WLM). This study examined dietary quality and micronutrient adequacy among WLMs in a commercial weight management program. Participants were 1207 WLM in Weight Watchers (WW) who had maintained a 9.1 kg or greater weight loss (29.7 kg on average) for 3.4 years and had a body mass index (BMI) of 28.3 kg/m^2^. A control group of weight stable adults with obesity (controls; N = 102) had a BMI of 41.1 kg/m^2^. Measures included the Diet History Questionnaire-II, Healthy Eating Index-2015 (HEI), and Dietary References Intakes. WLM versus controls had a 10.1 point higher HEI score (70.2 (69.7–70.7) vs. 60.1 (58.4–61.8); *p* = 0.0001) and greater odds of meeting recommendations for copper (OR = 5.8 (2.6–13.1)), magnesium (OR = 2.9 (1.8–4.7)), potassium (OR = 4.7 (1.4–16.5)), vitamin A (OR = 2.8 (1.7–4.8)), vitamin B6 (OR = 2.9 (1.6–5.2)), and vitamin C (OR = 5.0 (2.8–8.8)). WLM, compared to controls, also reported higher percentages of calories from carbohydrates (50.3% (49.7–50.8) vs. 46.7% (44.8–48.7); *p* = 0.0001) and protein (18.2% (18.0–18.5) vs. 15.9% (15.1–16.6); *p* = 0.0001) and lower calories from fat (32.3% (31.9–32.8) vs. 37.4% (35.8–38.9); *p* = 0.0001). Long-term weight loss maintenance in a widely used commercial program was associated with a healthier diet pattern, including consuming foods with higher micronutrient density.

## 1. Introduction

Many individuals with overweight or obesity exceed energy needs but do not meet vitamin and mineral requirements [1,2]. Individuals with obesity are at risk for several micronutrient inadequacies, including inadequate iron, calcium, magnesium, zinc, copper, folate, and vitamins A, B_12_, C, D, and E [1,2]. Poor diet quality, including limited fruits and vegetables, beans, and whole grains, and overconsumption of high-calorie, low nutrient value foods and added sugars are considered to be the major contributors to nutrient inadequacies in the US [1,2,3,4,5]. Poor diet quality and related micronutrient inadequacies may increase risk of several chronic diseases, including cancer, cardiovascular disease, type 2 diabetes, and osteoporosis, and have been linked to other symptoms, including increased fatigue, reduced ability to fight infections, and impaired cognitive function (i.e., attention, memory, and mood) [1,2,6,7]. Obesity is also linked to—and may compound—these disease and health risks [8].

Strategies to reduce micronutrient inadequacies include food enrichment and fortification and the use of multivitamin and mineral supplements. These strategies have reduced, but not removed, inadequate micronutrient intake [9], and the use of supplements to prevent chronic disease remain controversial, given the inconsistent results [10]. Weight loss programs, designed to promote healthy eating and reduce excess calorie intake, have been found to result in improved diet quality [11,12,13] and micronutrient status in some studies [14,15]. Commercial weight loss programs that reach a large segment of the US population have also been shown to promote improvements in diet quality [16,17,18]. Whether improved diet quality and micronutrient density is characteristic of individuals with sustained long-term weight loss maintenance is unknown [13,19].

The primary aim of this study was to quantify the dietary intake of long-term, successful weight loss maintainers who had lost at least 20 lbs and maintained that loss for at least one year in a widely available commercial weight loss program. WW (formerly Weight Watchers) is designed to encourage a diet that is lower in saturated fat and sugar, controls energy intake dependent on weight management goals, and shifts towards frequent consumption of lean proteins, non-fat dairy, and fruits and vegetables [20,21,22,23]. In prior research, WW produced clinically significant average long-term weight loss [20,21,22,23] and modest increases in fruit and vegetable intake [17,18]. In the current study, we hypothesized that, compared to weight stable individuals with obesity, long-term successful weight loss maintainers in WW would have improved overall diet quality (measured by the Health Eating Index-2015) and increased the odds of meeting national recommendations for vitamin and mineral adequacy.

## 2. Materials and Methods 

### 2.1. Study Design and Participants

The WW Success Registry is a cross-sectional observational study of individuals who lost weight in the WW program and were successful at long-term (≥1 year) maintenance of ≥20 lb (9.1 kg) weight loss. Weight-stable individuals were enrolled as a control group to distinguish the factors associated with successful maintenance weight loss vs. weight stable obesity. The study was conducted at California Polytechnic State University (Cal Poly) and the procedures were approved by the Cal Poly Institutional Review Board (ethical approval code 2018-022). All participants provided informed consent electronically via Research Electronic Data Capture (RedCap).

Weight loss maintainers: To be eligible for enrollment, individuals must have been aged >18 years and had maintained a >20 lb (9.1 kg) loss from WW entry for >1 year. The 20 lb (9.1 kg) criterion was selected to approximate a clinically significant 10% weight loss [24], assuming a 200 lb (90 kg) starting weight among people entering WW and other weight loss programs [25]. Specific BMI criteria at WW entry were not required, but the average BMI of people entering WW has been ~30 kg/m^2^ [18]. Prospective weight loss maintainers were recruited through an email sent by WW to members who had reported a loss in WW of ≥20 lb more than 1 year before. Interested individuals were referred to the study website hosted by Cal Poly for online screening, consent, and enrollment. After completion of the survey, participants received an electronic summary of their macronutrient intake.

Weight stable individuals with obesity (“controls”): To be eligible for enrollment, individuals must have been aged >18 years, had a BMI >30 kg/m^2^ and reported weight stability (5 lbs) for >5 years prior to enrollment [26]. Weight stable individuals with obesity were recruited through local and national advertising channels, including Facebook, ResearchMatch.org, Amazon, Mechanical Turk, and via the Cal Poly Center for Health Research registry; consenting and assessment procedures were the same in both groups. After completion of the survey, participants were provided one month of the WW online program (WW Digital), free of charge, and also received an electronic summary of their macronutrient intake.

### 2.2. Measures

All measures were administered online via RedCap immediately after consent. Participants were asked standard demographic information (age, education level, and marital status), current weight and height, and details about weight history (age of onset of overweight and maximum lifetime weight) [27,28].

The Diet History Questionnaire (DHQ-II) from the National Institutes of Health was used to measure dietary intake [29]. The DHQ-II assesses dietary intake over the past 12 months for 134 food items and includes questions regarding portion sizes and intakes of vitamins, minerals, and herbal supplements [29]. The nutrient and food group database for analyzing the DHQ-II is based on 24 h recall data from the National Health and Nutrition Examination Surveys (NHANES). The NHANES data were analyzed to provide a single nutrient or food group value by portion size for each food on the DHQ II. The primary databases used to compute nutrient and food group estimates are the U.S. Department of Agriculture’s (USDA) Food and Nutrient Database for Dietary Studies (FNDDS) and USDA’s MyPyramid Equivalents Database [29,30].

Since the DHQ-II asks about usual intake over the past year, individuals reporting biologically implausible daily energy intakes were removed from the analysis. The cut offs selected for this study were based on previous population studies that analyzed food frequency questionnaire data in which individuals who reported implausible daily energy intakes were excluded from the analysis; exclusions were based on less than 800 kcals per day or more than 4200 kcals per day for men, and less than 600 kcals per day or more than 3500 kcals per day for women [30]. In this study, 44 individuals (38 weight loss maintainers and 6 controls) were removed for implausible values based on these criteria.

The diet quality of the individuals in the study was computed using the Healthy Eating Index-2015. The Healthy Eating Index-2015 reflects adherence to the U.S. government’s recommended 2015–2020 Dietary Guidelines for Americans and has demonstrated both construction validity and reliability as a diet quality measure between groups [31]. The components for scoring a result in at highest possible score of 100 and to quantify adequate intake for a total of 13 categories, including: Total fruits, whole fruits, total vegetables, greens and beans, whole grains, dairy, total protein foods, seafood and plant proteins, fatty acids and moderation in refined grains, sodium, added sugars, and saturated fats (Supplementary Table S1) [32].

The dietary reference intake for vitamins and minerals was used to determine the proportion of individuals whose diets met the estimated average requirement (EAR) using the cut-point method [33]. The cut-points were based on age and sex and are listed for each vitamin in Supplementary Tables S2 and S3. Vitamins and minerals in which an EAR has not been established were excluded with the exception of potassium, since it has been shown in the literature that adults with obesity are more likely to have diets that are deficient in potassium [2]. For potassium, we applied the adequate intake (AI) cut-point. Molybdenum and iodine were also excluded because these minerals were not measured in the DHQ-II.

### 2.3. Statistics

Independent *t*-tests and chi-square analyses were conducted to examine differences among adults who completed vs. did not complete the DHQ-II and the differences between weight loss maintainers and controls. General linear models (for Healthy Eating Index-2015 scores and macronutrients) and logistic regression models (for EAR cutoffs) were used to examine dietary differences between the weight loss maintainers and controls on dependent measures, while controlling for the following demographic characteristics: Age, sex, income, education, employment, race, marital status, and maximum lifetime weight (kg). Discriminant function analysis was used to determine the dietary factors that most discriminated weight loss maintainers from weight stable individuals with obesity among the set of variables that were found to differ between the two groups in the general linear and logistic regression analyses. The resulting standardized canonical coefficients represent the measure of association between the discriminant function (based on the linear combination of variables) and each dietary variable and indicate the relative strength of each variable in distinguishing the two groups (similar to a beta weights in a multiple regression). To reduce the probability of committing false statistical inferences, a Bonferroni correction was used to adjust for 19 multiple comparisons (18 micronutrients and 1 Healthy Eating Index-2015 analysis), and a *p*-value of less than 0.003 was then set to indicate statistical significance. The SPSS (23.0.0) statistical package was used for all analyses.

## 3. Results

### 3.1. Characteristics of Weight Loss Maintainers versus Controls

Demographic characteristics of the 1309 participants who completed the DHQ-II are presented in Table 1. The weight loss maintainers were more likely than controls to be older, have a lower current BMI and lifetime maximum weight, to have a higher total family income >$75,000, and to be Caucasian and married.

### 3.2. Group Differences in Healthy Eating Index and Macronutrients

**Figure 1:**
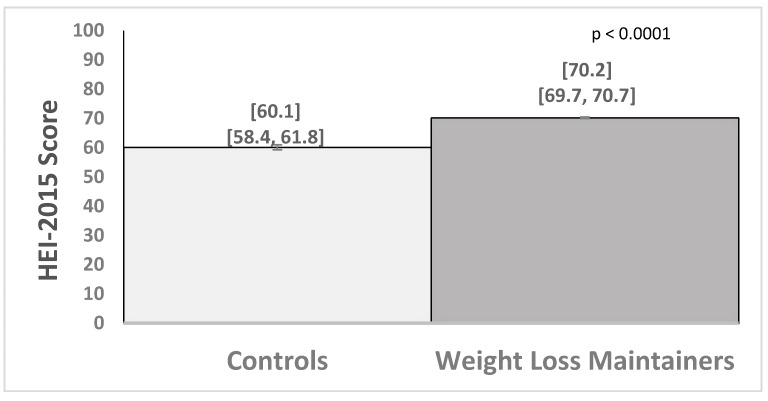
Average (95% confidence interval) Healthy Eating Index-2015 scores for controls (N = 102) and weight loss maintainers (N = 1207).

**Figure 2:**
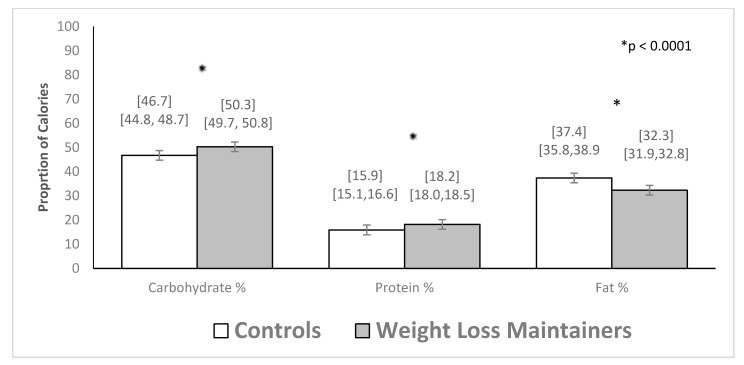
Average (95% confidence interval) macronutrient intake among controls (N = 102) and weight loss maintainers (N = 1207). * denotes a statistically significant difference between the control and weight loss maintainer groups at the *p* < 0.0001 level

In analyses that adjusted for group differences in demographic factors, weight loss maintainers had significantly higher average Healthy Eating Index-2015 scores compared to controls (Figure 1). The Healthy Eating Index-2015 score was 10.1 points (95% Confidence Interval (CI), 8.3,11.9) higher on average for weight loss maintainers than controls. The total average energy intake in kcals did not significantly differ between weight loss maintainers and controls (1502 (1470,1533) vs. 1552 (1445,1659) kcal/day, respectively; B = −51.9 (−165.7,61.8); *p* = 0.37). However, the percentage of calories from the macronutrients in the diet (carbohydrate, protein and fat) differed between the groups (Figure 2). Examining food groups, weight loss maintainers versus controls had significantly more daily cup equivalents of fruit (2.3 (2.2, 2.3) vs. 1.1 (0.8, 1.4); *p* = 0.0001) and vegetables (2.3 (2.2, 2.3) vs. 1.7 (1.4, 2.0); *p* = 0.001), and did not significantly differ in daily ounce equivalents of lean meat (3.9 (3.7, 4.0) vs. 3.1 (2.6, 3.6)); *p* = 0.003), cup equivalents of milk (1.2 (1.1, 1.4) vs. 1.0 (0.9, 1.1); *p* = 0.01) or ounce equivalents of grain (3.4 (3.3, 3.5) vs. 3.7 (3.3, 4.1); *p* = 0.09).

### 3.3. Group Differences in Proportions Meeting Estimated Average Requirement for Micronutrients

Examining the proportion of weight loss maintainers and controls that met the estimated average requirement (EAR) for micronutrients revealed significant differences between the two groups (Table 2 and Table 3). Weight loss maintainers from dietary sources alone and excluding supplement intake were significantly more likely to meet the EAR for copper, riboflavin, vitamin A, and vitamin C compared to controls. Weight loss maintainers had 7.6 times higher odds for meeting the EAR for copper (OR = 7.6 (95% CI 3.7,15.3); *p* = 0.0001) and 4–5 times higher odds for riboflavin (OR = 5.3 (2.1,13.5); *p* = 0.0001), and vitamin C (OR = 4.3 (2.6,7.0); *p* = 0.0001) and 2 times higher odds for vitamin A (OR = 2.3 (1.4,3.7); *p* = 0.001). Trends were observed for magnesium (OR = 1.7 (1.1,2.7); *p* = 0.017), potassium (OR = 6.8 (1.5,30.5); *p* = 0.012), thiamin (OR = 1.7 (1.1,2.8); *p* = 0.026), and folate (OR = 1.7 (1.1,2.7); *p* = 0.020).

The majority of participants in both groups reported taking dietary supplements over the past year (Table 1). An analysis of group differences in micronutrient intake from both diet and supplements revealed similar findings (Table 3). For all micronutrients and among both weight loss maintainers and controls, the inclusion of supplement use resulted in an increased proportion of individuals meeting the EAR compared to diet alone. In Table 2 and Table 3, this was especially apparent in meeting the EAR for vitamin D (weight loss maintainers diet only: 5.3% vs. diet plus supplements: 54.0% and controls 4.2% vs. 42.2%), vitamin E (weight loss maintainers 11.1% vs. 61.2% and controls 11.8% vs. 46.1%), calcium (weight loss maintainers 32.1% vs. 54.4% controls 42.2% vs. 56.9%), and iron (weight loss maintainers 78.1% vs. 96.3% and controls 75.5% vs. 91.2%).

### 3.4. Multiple Discriminant Analysis

Multiple discriminant analysis was conducted to determine, among the dietary variables that differed between groups, which ones most strongly and independently discriminated weight loss maintainers from weight stable individuals with obesity. Standardized canonical coefficients indicated that Healthy Eating Index scores (0.59) and meeting the EARs for vitamin C (0.20) and copper (0.18) contributed independently and the most (48.5% of variance; *p* = 0.0001) to discriminating the two groups. 

## 4. Discussion

Compared with weight stable individuals with obesity, weight loss maintainers in a widely used commercial weight management program, who had maintained successful weight loss for over three years on average, had higher Healthy Eating Index-2015 scores, suggesting a greater intake of fruits, vegetables, beans, and whole grains, and less refined foods, sodium, added sugars, and saturated fats. Weight loss maintainers, relative to controls, also had higher odds of meeting recommendations for several micronutrients, including seven times higher odds of meeting recommendations for copper, four times higher odds for vitamin C, and twice the odds for meeting recommended levels of magnesium, vitamin A, riboflavin, and vitamin B6. Thus, long-term weight loss maintenance was associated with a healthier diet pattern, including consuming foods with higher micronutrient density.

This study is the first to examine Health Eating Index-2015 scores among long-term weight loss maintainers and in a non-clinic, widely used commercial weight management program. The higher Healthy Eating Index-2015 scores among weight loss maintainers were consistent with the WW program’s emphasis on higher quality, nutrient-dense foods and prior evaluation of the program’s dietary prescription [16]. Short-term (≤12 month) clinical trials have found that WW increased intake of fruit and vegetables [17] fiber and protein [18] and reduced the total and saturated fat intake [18]. Outside of WW, other behavioral weight loss trials that targeted healthy eating, activity, and behavioral strategies have also found significant increases in Healthy Eating Index-2015 scores in association with weight loss over four–six months [11,12,13,34]. One 18 month study of weight loss and maintenance with portion controlled meals found that Healthy Eating Index-2010 scores increased initially by 20.3 points over six months, then moderated to an overall 11.3 increase at 18 months [13]. In the current study, among weight loss maintainers who had maintained their weight loss for 3.4 years, a similar magnitude, 10-point increase, relative to weight stable individuals with obesity, was observed. However future prospective research is needed to examine whether and how improved Healthy Eating Index scores change over time and support weight loss maintenance. 

In the analysis of micronutrient intake from both diet and supplements, weight loss maintainers met the recommendations for the majority of micronutrients and were significantly more likely than weight stable individuals with obesity to meet the estimated average requirements for copper, magnesium, vitamin A, riboflavin, vitamin B6, and vitamin C. In an earlier study, female weight loss maintainers in the National Weight Control Registry were found to have significantly higher intakes of vitamins A and C and calcium compared to adult women in NHANES III [19]. Improved micronutrient intake and lower body mass might both serve to reduce disease risks among weight loss maintainers. Adequate intake of antioxidants such as vitamins A, C, and E has been associated with lower risk of atherosclerosis and improved glucose metabolism and insulin resistance [35]. Dietary intake of adequate B-vitamins has been shown to be important in cognitive function and is associated with a decreased risk of dementia and depression [36]. Higher magnesium has been associated with decreased risk of cardiovascular disease, ischemic heart disease mortality [35,37], incidence of diabetes, and lower bone density [35]. Future research may seek to quantify the extent to which weight loss maintainers have improved health outcomes due to sustained improvement in diet quality, lower weight, or a synergistic effect [7]. Additionally, measuring blood levels of micronutrients would provide clinical evidence of micronutrient sufficiency in the diet.

Notably, while most micronutrients were improved among weight loss maintainers relative to weight stable individuals with obesity, weight loss maintainers remained inadequate in some nutrients. Most (90%) weight loss maintainers did not meet adequate intake for potassium, and about 50% did not meet the recommendations for calcium and vitamin D; 20%–30% did not meet recommendations for magnesium and vitamin E. These proportions are generally better than US population averages [2]. Future weight management interventions and WW may seek ways to alter dietary advice concerning foods rich in these insufficient micronutrients in order to improve intake among weight loss maintainers. 

The majority of both weight loss maintainers (76.6%) and weight stable individuals with obesity (68.6%) reported taking supplements over the past year. In the general population from NHANES 2011–2012, 52% of US adults took at least one dietary supplement in the past 30 days [38]. The proportions of weight loss maintainers and weight stable individuals with obesity that met the EAR for micronutrients increased as a result of including supplements. However, recommending multivitamin and mineral supplements remains controversial since past research focused on their health effects have yielded inconsistent results [10]. The Dietary Guidelines for Americans 2015–2020 recommended getting the majority of micronutrients from food sources [39,40].

Weight loss maintainers compared with weight stable individuals with obesity had a modestly higher average percentage of calories from protein and carbohydrates and a significantly lower average percentage of calories from fat. In a systematic review of determinants of weight loss maintenance, the only macronutrient that was predictive of successful weight maintenance was a decrease in fat intake, while intake of carbohydrate or protein failed to show conclusive evidence [41]. In weight loss maintainers in the National Weight Control Registry, the percentage of calories from fat in the diet was considerably lower than among weight loss maintainers in the current study (24.3 vs. 32.3%, respectively). However, fat intake in the National Weight Control Registry has increased over time as the popularity of low fat diets has subsided [42]. The 2015–2020 Dietary Guidelines for Americans [39,40] recommended that the percentage of calories from fat in the diet range from 20% to 35% of total calories, consistent with the range of calories from fat reported among weight loss maintainers in the current study. The weight stable individuals with obesity group reported consuming 37% calories from fat, which exceeded this recommended range. 

This study has several strengths and some limitations. The study is one of the first to examine dietary quality and micronutrient adequacy using a validated diet history questionnaire (DHQ-II) among weight loss maintainers outside the clinic setting in a widely used commercial weight management program. Dietary analysis was conducted for over 1200 long-term weight loss maintainers and included a comparison group of more than 100 weight stable individuals with obesity. However, the dietary and supplement intake were based solely on self-reporting, which is vulnerable to underreporting bias, particularly among individuals with higher BMIs [43,44]. While extreme underreporters were excluded, micronutrient intake and adequacy could remain underestimated in both groups and particularly among controls. Future research measuring blood levels of micronutrients would provide clinical evidence of micronutrient sufficiency in the diet. The current study did not examine proportions exceeding the upper limits of recommended micronutrients. The DHQ-II is comprehensive in capturing the dietary intake over a year; however, only 17% of participants in the WW Success Survey study completed the DHQ-II. Those who chose to complete the DHQ-II differed from non-completers in age, race, and employment. Factors that might have reduced completion rates of the DHQ-II included it taking about an hour to complete and needing to be completed on an external website at the end of a lengthy survey. There were also significant differences in the demographic characteristics of the weight loss maintainers and weight stable individuals with obesity. In particular, weight loss maintainers tended to be older and a higher percentage were female. Eligibility criteria were based on self-report, and participants were self-selected; thus, findings may not generalize to the population at large. Several analyses of one dataset could inflate Type 1 error, but Bonferroni correction was used to guard against this risk. A cross sectional design was used; thus, causality cannot be inferred.

In conclusion, long-term weight loss maintenance was associated with greater odds of meeting micronutrient recommendations and consumption of a nutrient dense diet. Future research is needed to determine the mechanisms linking improved micronutrient density with long-term, successful weight loss maintenance. 

## Figures and Tables

**Table 1:** Characteristics of weight loss maintainers (WLM; N = 1207) versus controls (N = 102).

	Controls	WLM	*p* Value
	N = 102	N = 1207	
Age, years, mean (SD)	48.6 (13.2)	55.6 (12.3)	0.0001
Female, %	82.4	91.4	0.003
Currently in WW, %	5.4	92.2	0.0001
Lifetime maximum weight, kg	124.5 (29.7)	105.0 (22.1)	0.0001
Weight at start of WW, kg	—	100.9 (20.9)	
BMI at start of WW, kg/m^2^	—	36.5 (7.0)	
Current weight, kg	113.2 (24.3)	75.2 (15.2)	0.0001
Weight loss since WW start, kg	—	25.7 (13.2)	
Duration of 9.1 kg loss from WW start, years	—	3.4 (3.0)	
Weight lost from maximum weight, kg	11.3 (13.2)	29.7 (15.0)	0.0001
Current BMI, kg/m^2^	41.1 (8.8)	27.2 (5.1)	0.0001
BMI Categories			0.0001
Obese, %	100	19.4	
Overweight, %	0	45.4	
Normal weight, %	0	35.2	
Underweight, %	0	0	
Income (total in family per year)			0.0001
<$25,000	16.8	4.3	
$25,001–75,000	52.5	31.6	
>$75,000	30.7	64.0	
Race/ethnicity			
White, %	84.3	94.9	0.0001
Black, %	13.7	2.2	0.0001
Hispanic, %	3.9	3.4	0.773
Employed, %	72.5	62.5	0.043
College education or more, %	88.2	90.1	0.794
Married, %	55.0	74.2	0.0001
Supplement use ^a^, %	68.6	76.6	0.069

^a^ Supplement use was defined as the use at least one micronutrient supplement which included either individual micronutrient supplements or supplements which provide multiple vitamins and minerals. Few (*n* = 9 WLM) participants reported current use of medications for weight management.

**Table 2:** Proportion of weight loss maintainers and controls meeting the estimated average requirement for minerals based on age and sex.

		Controls	WLM	Chi-Square ^a^	Odds Ratio ^b^
		(N = 102)	(N = 1207)	*p*-Value	95% CI
**Calcium**	Diet	42.2%	32.1%	0.037 *	0.92 (0.58–1.47); *p* = 0.726
	Diet + Suppl	56.9%	54.4%	0.636	1.05 (0.67–1.65); *p* = 0.823
**Copper**	Diet	81.4%	96.2%	0.0001 *	7.56 (3.74–15.32); *p* = 0.0001 *
	Diet + Suppl	87.3%	97.2%	0.0001 *	5.84 (2.60–13.11); *p* = 0.0001 *
**Iron**	Diet	75.5%	78.1%	0.537	1.47 (0.86–2.51); *p* = 0.159
	Diet + Suppl	91.2%	96.3%	0.013 *	1.91 (0.79–4.62); *p* = 0.151
**Magnesium**	Diet	49.0%	62.1%	0.010 *	1.73 (1.10–2.72); *p* = 0.017
	Diet + Suppl	61.8%	81.4%	0.0001 *	2.87 (1.77–4.65); *p* = 0.0001 *
**Phosphorus**	Diet	89.2%	93.5%	0.096	1.54 (0.72–3.32); *p* = 0.269
	Diet + Suppl	90.2%	95.6%	0.014 *	1.97 (0.88–4.45); *p* = 0.101
**Potassium ^c^**	Diet	2.0%	9.6%	0.010 *	6.83 (1.53–30.48); *p* = 0.012
	Diet + Suppl	2.9%	10.1%	0.018 *	4.72 (1.35–16.47); *p* = 0.015
**Selenium**	Diet	90.2%	91.6%	0.617	1.14 (0.53–2.48); *p* = 0.739
	Diet + Suppl	92.2%	95.6%	0.112	1.56 (0.64–3.81); *p* = 0.332
**Zinc**	Diet	70.6%	72.9%	0.613	1.21 (0.74–2.00); *p* = 0.447
	Diet + Suppl	81.4%	87.3%	0.088	1.66 (0.92–3.01); *p* = 0.095

WLM = Weight loss maintainers; * statistically significant difference between the control and weight loss maintainer groups ^a^ Proportions were unadjusted. ^b^ Odds ratio adjusted for group, age, sex, employment, education, marital status, race, income, and maximum weight in kg. ^c^ The adequate intake cut-point was used for potassium. Potassium does not have an estimated average requirement.

**Table 3:** Proportion of weight loss maintainers and controls meeting the estimated average requirement for vitamins based on age and sex.

		Control (N = 102)	WLM (N = 1207)	Chi-sq *p*-Value ^a^	Odds Ratio (95% CI) ^b^
**Vitamin A**	Diet	48.0%	71.4%	0.0001 *	2.28 (1.42–3.66); *p* = 0.001 *
	Diet + Suppl	69.6%	85.9%	0.0001 *	2.82 (1.68–4.75); *p* = 0.0001 *
**Thiamin (B1)**	Diet	64.7%	73.9%	0.044 *	1.72 (1.07–2.78); *p* = 0.026
	Diet + Suppl	77.5%	88.1%	0.002 *	2.33 (1.33– 4.09); *p* = 0.003
**Riboflavin (B2)**	Diet	89.2%	98.0%	0.0001 *	5.33 (2.11–13.5); *p* = 0.0001 *
	Diet + Suppl	92.2%	99.0%	0.0001 *	6.54 (2.21–19.34); *p* = 0.001 *
**Niacin (B3)**	Diet	82.4%	87.4%	0.145	1.64 (0.90–2.98); *p* = 0.110
	Diet + Suppl	90.2%	94.7%	0.059	1.85 (0.82–4.14); *p* = 0.136
**Vitamin B6**	Diet	65.7%	76.6%	0.014 *	2.29 (1.41–3.73); *p* = 0.001 *
	Diet + Suppl	79.4%	88.9%	0.004 *	2.91 (1.61–5.24); *p* = 0.0001 *
**Vitamin B12**	Diet	85.3%	86.6%	0.716	1.12 (0.58–2.13); *p* = 0.741
	Diet + Suppl	92.2%	93.5%	0.613	1.23 (0.53–2.84); *p* = 0.625
**Vitamin C**	Diet	56.9%	85.3%	0.0001 *	4.29 (2.63–7.00); *p* = 0.0001 *
	Diet + Suppl	71.6%	92.7%	0.0001 *	4.97 (2.80–8.80); *p* = 0.0001 *
**Vitamin D**	Diet	4.9%	5.3%	0.862	1.22 (0.44–3.44); *p* = 0.704
	Diet + Suppl	42.2%	54.0%	0.021 *	1.49 (0.94–2.35); *p* = 0.090
**Vitamin E**	Diet	11.8%	11.1%	0.838	0.87 (0.44–1.72); *p* = 0.680
	Diet + Suppl	46.1%	61.2%	0.003 *	1.76 (1.12–2.77); *p* = 0.014
**Folate**	Diet	53.9%	63.6%	0.051	1.72 (1.09–2.72); *p* = 0.020
	Diet + Suppl	70.6%	83.1%	0.002 *	2.20 (1.32–3.68); *p* = 0.003

WLM = Weight loss maintainers; * statistically significant difference between the control and weight loss maintainer groups; ^a^ Proportions were unadjusted; ^b^ Odds ratio adjusted for group, age, sex, employment, education, marital status, race, income, and maximum weight in kg.

## Supplementary Materials

The following are available online at https://www.mdpi.com/2072-6643/11/12/3046/s1, Table S1: Healthy Eating Index-2015 Scoring. Table S2: Vitamin Dietary Reference Intakes and Estimated Average Requirement from the Food and Nutrition Board, National Academies of Sciences, Engineering and Medicine. Table S3: Mineral Dietary Reference Intake: Estimated Average Requirement, Food and Nutrition Board, National Academies of Sciences, Engineering, and Medicine
