# Supplementary material for: Diet Quality and Micronutrient Intake among Long-Term Weight Loss Maintainers
Source: Nutrients. 2019 Dec 13;11(12):3046. doi: 10.3390/nu11123046 (PMC6950482; doi:10.3390/nu11123046)
Supplement: Supplementary file 1 [file nutrients-11-03046-s001.pdf]

Supplementary Table S1: Healthy Eating Index-2015 Scoring.

Adequacy Components	Total Possible Points	Standard to Maximize Points	Standard for score of zero
Total Fruits	5	≥0.8 cup equivalents/1,000 kcal	No fruit
Whole Fruits	5	≥0.4 cup equivalents/1,000 kcal	No whole fruit
Total Vegetables	5	≥1.1 cup equivalents/1,000 kcal	No vegetables
Greens and Beans	5	≥0.2 cup equivalents/1,000 kcal	No dark green veg/beans/peas
Whole Grains	10	≥1.5 oz. equivalents/1,000 kcal	No whole grains
Dairy	10	≥1.3 cup equivalents/1,000 kcal	No dairy
Total Protein Foods	5	≥2.5 oz. equivalents/1,000 kcal	No protein foods
Seafood & Plant Protein	5	≥0.8 oz. equivalents/1,000 kcal	No seafood/plant proteins
Fatty Acids	10	(PUFA+MUFA)/SFA ≥ 2.5	(PUFA+MUFA)/SFA ≤ 1.2
**Moderation Components**			
Refined Grains	10	≤ 1.8 oz. equivalents/1,000 kcal	≥4.3 oz. equivalents/1,000 kcal
Sodium	10	≤ 1.1 g/1,000 kcal	≥ 2.0 g/1,000 kcal
Added Sugars	10	≤ 6.5% energy	≥ 26% of energy
Saturated Fats	10	≤ 8% energy	≥ 16% of energy
**Maximum Possible Score**	100		

Source: Reedy et al., (2018)[31]; PUFA: polyunsaturated fatty acids; MUFA: monounsaturated fatty acids; SFA: saturated fatty acids.

Supplementary Table S2: Reference Intakes: Estimated Average Requirement from the Food and Nutrition Board, National Academies of Sciences, Engineering and Medicine.

		Males		Females	
		19–50 y	> 51 y	19–50 y	>51 years
Vit A	(µg/d)[a]	625	625	500	500
Vit C	(mg/d)	75	75	60	60
Vit D	(µg/d)[b]	10	10	10	10
Vit E	(mg/d)[c]	12	12	12	12
Thiamin	(mg/d)	1	1	0.9	0.9
Riboflavin	(mg/d)	1.1	1.1	0.9	0.9
Niacin	(mg/d)[d]	12	12	11	11
Vit B6	(mg/d)	1.1	1.4	1.1	1.3
Folate	(µg/d)[e]	320	320	320	320
Vit B12	(µg/d)	2	2	2	2

Source: https://www.ncbi.nlm.nih.gov/books/NBK56068/table/summarytables.t1/?report=objectonly; The data in this table was taken from the DRI reports (available at www.nap.edu). An Estimated Average Requirement (EAR) is the average daily nutrient intake level estimated to meet the requirements of half of the healthy individuals in a group. EARs have not been established for Vitamin K, pantothenic acid, biotin choline, chromium, flouride, manganese, potassium, sodium, or chloride. SOURCES: DRI for Calcium, Phosphorous, Magnesium, Vitamin D, and Fluoride (1997); DRIs for Thiamin, Riboflavin, Niacin, Vitamin B6, Folate, Vitamin B12, Pantothenic Acid, Biotin, and Choline (1998); DRIs for Vitamin C, Vitamin E, Selenium, and Carotenoids (2000); DRIs for Vitamin A, Vitamin K, Arsenic, Boron, Chromium, Copper, Iodine, Iron, Manganese, Molybdenum, Nickel, Silicon, Vanadium, and Zinc (2001); DRIs for Energy, Carbohydrate, Fiber, Fat, Fatty Acids, Cholesterol, Protein, and Amino Acids (2002/2005); and Water, Potassium, Sodium, Chloride, and Sulfate (2005); and DRIs for Calcium and Vitamin D (2011). These reports may be accessed via www.nap.edu. [a] As retinol activity equivalents (RAEs). 1 RAE = 1 µg retinol, 12 µg β-carotene, 24 µg α-carotene, or 24 µg β-cryptoxanthin. The RAE for dietary provitamin A carotenoids is two-fold greater than retinol equivalents (REs), whereas the RAE for preformed vitamin A is the same as RE. [b] As cholecalciferol. 1 µg cholecalciferol = 40 IU vitamin D. Assumes minimal sun exposure. [c] As α-tocopherol. α-tocopherol includes RRR-α-tocopherol, the only form of α-tocopherol that occurs naturally in foods, and the 2R-stereoisomeric forms of α-tocopherol (RRR-, RSR-, RRS-, and RSS-α-tocopherol) that occur in fortified foods and supplements. It does not include the 2S-stereoisomeric forms of α-tocopherol (SRR-, SSR-, SRS-, and SSS-α-tocopherol), also found in fortified foods and supplements. [d] As niacin equivalents (NE). 1 mg of niacin = 60 mg of tryptophan. [e] As dietary folate equivalents (DFE). 1 DFE = 1 µg food folate = 0.6 µg of folic acid from fortified food or as a supplement consumed with food = 0.5 µg of a supplement taken on an empty stomach.

Supplementary Table S3: Mineral Dietary Reference Intake: Estimated Average Requirement, Food and Nutrition Board, National Academies of Sciences, Engineering, and Medicine.

		Males		Females		
		**19–70 y**	**> 70 y**	**19–30 y**	**31–50 y**	**>50**
**Calcium**	(mg/d)	800	1000	800	800	1000
**Copper**	(μg/d)	700	700	700	700	700
**Iodine**	(μg/d)	95	95	95	95	95
**Iron**	(mg/d)	6	6	8.1	8.1	5
**Magnesium**	(mg/d)	330	330	255	265	265
**Phosphorus**	(mg/d)	580	580	580	580	580
**Potassium[a]**	(g/d)	4.7	4.7	4.7	4.7	4.7
**Selenium**	(μg/d)	45	45	45	45	45
**Zinc**	(mg/d)	9.4	9.4	6.8	6.8	6.8

Source: https://www.ncbi.nlm.nih.gov/books/NBK56068/table/summarytables.t1/?report=objectonly; [a] Potassium has an AI rather than an EAR established. The data in this table was taken from the DRI reports (available at www.nap.edu). An EAR is the average daily nutrient intake level estimated to meet the requirements of half of the healthy individuals in a group. EARs have not been established for vitamin K, pantothenic acid, biotin choline, chromium, flouride, manganese, potassium, sodium, or chloride. SOURCES: *DRIs for Calcium, Phosphorous, Magnesium, Vitamin D, and Fluoride* (1997); *DRIs for Thiamin, Riboflavin, Niacin, Vitamin* B_6, *Folate, Vitamin* B_{12}, *Pantothenic Acid, Biotin, and Choline* (1998); *DRIs for Vitamin C, Vitamin E, Selenium, and Carotenoids* (2000); *DRIs for Vitamin A, Vitamin K, Arsenic, Boron, Chromium, Copper, Iodine, Iron, Manganese, Molybdenum, Nickel, Silicon, Vanadium, and Zinc* (2001); *DRIs for Energy, Carbohydrate, Fiber, Fat, Fatty Acids, Cholesterol, Protein, and Amino Acids (2002/2005); and Water, Potassium, Sodium, Chloride, and Sulfate* (2005); and *DRIs for Calcium and Vitamin D* (2011). These reports may be accessed via www .nap.edu.
